# SIRT1 and SIRT2: emerging targets in neurodegeneration

**DOI:** 10.1002/emmm.201302451

**Published:** 2013-02-18

**Authors:** Gizem Donmez, Tiago F Outeiro

**Affiliations:** 1 Department of Neuroscience, Tufts University School of MedicineBoston, Massachusetts, USA; 2 Department of Neurodegeneration and Restorative Research, Center for Nanoscale Microscopy and Molecular Physiology of the Brain, University Medical CenterGöttingen, Göttingen, Germany

**Keywords:** aging, Alzheimer's disease, neurodegeneration, Parkinson's disease, sirtuins

## Abstract

Sirtuins are NAD-dependent protein deacetylases known to have protective effects against age-related diseases such as cancer, diabetes, cardiovascular and neurodegenerative diseases. In mammals, there are seven sirtuins (SIRT1-7), which display diversity in subcellular localization and function. While SIRT1 has been extensively investigated due to its initial connection with lifespan extension and involvement in calorie restriction, important biological and therapeutic roles of other sirtuins have only recently been recognized. Here, we review the potential roles and effects of SIRT1 and SIRT2 in neurodegenerative diseases. We discuss different functions and targets of SIRT1 and SIRT2 in a variety of neurodegenerative diseases including Alzheimer's disease (AD), Parkinson's disease (PD) and Huntington's Disease (HD). We also cover the role of SIRT1 in neuronal differentiation due to the possible implications in neurodegenerative conditions, and conclude with an outlook on the potential therapeutic value of SIRT1 and SIRT2 in these disorders.

## Sirtuins, calorie restriction and aging

Sirtuins, or silent information regulator 2 (SIR2) proteins, are enzymes present in organisms ranging from bacteria to plants, and animals (Haigis & Sinclair, 2010). Initial studies in the budding yeast *Saccharomyces cerevisiae* led to the discovery that extra copies of the *SIR2* gene extended lifespan by 50%, whereas *SIR2* deletion reduced longevity (Kaeberlein et al, 1999). *SIR2* promotes longevity in yeast by suppressing the formation of toxic extra-chromosomal rDNA circles (ERCs; Sinclair & Guarente, 1997). Subsequent studies demonstrated that in worms and flies *SIR2* orthologues also seemed to play a role in the regulation of life span (Tissenbaum & Guarente, 2001; Wood et al, 2004). However, the increase of longevity due to Sir2 overexpression in metazoans has recently been challenged and ascribed to genetic background differences, and is now considered to be smaller than initially proposed (Burnett et al, 2011; Viswanathan & Guarente, 2011). Nevertheless, several studies support that SIRT1, the closest mammalian orthologue of Sir2 in yeast, could be a critical mediator of the beneficial effects of calorie restriction (CR), a dietary regimen aimed at reducing calorie intake by 20–30% without malnutrition. CR has been shown to slow down the aging process and increase health- and lifespan in a variety of models tested (Austad, 1989, 2012; Bishop & Guarente, 2007; Jiang et al, 2000; Lakowski & Hekimi, 1998; Mair et al, 2003; Weindruch & Walford, 1982). Several studies reported that CR leads to increased levels of sirtuins in a variety of tissues (Cohen et al, 2004).

In mammals, there are seven sirtuins, SIRT1-7, all possessing a highly conserved central NAD^+^-binding site and common catalytic domain. Sirtuins are structurally different with respect to their N- and C-termini (Frye, 2000), their sub-cellular localization, and in that they utilize different substrates and protein binding partners [reviewed in (Guarente, 2011)]. With respect to sub-cellular distribution, SIRT1 is mainly nuclear, where it associates with euchromatin, although it can transiently be found in the cytoplasm (Tanno et al, 2007). SIRT6 is also a nuclear protein, associating with heterochromatin, and SIRT7 is nucleolar (Ford et al, 2006). SIRT2 is primarily cytosolic but it can also transiently shuttle into the nucleus in a cell cycle-dependent manner (North et al, 2003). SIRT3, SIRT4 and SIRT5 are mitochondrial proteins (Table 1; Michishita et al, 2005). Although some sirtuins may have redundant functions, their substrate specificity may also be influenced by intracellular compartmentalization, by different tissue expression patterns and distinct enzymatic activities.

**Table 1:** Mass, activity and localization of mammalian sirtuins

	Molecular Mass (kDa)	Enzymatic Activity	Sub-cellular Localization
SIRT1	81.7	Deacetylase	Nuclear, cytoplasmic
SIRT2	43.2	Deacetylase	Cytoplasmic, nuclear
SIRT3	43.6	Deacetylase	Mitochondrial
SIRT4	35.2	ADP-ribosyltransferase	Mitochondrial
SIRT5	33.9	Deacetylase	Mitochondrial
		Demalonylase	
		Desuccinylase	
SIRT6	39.1	ADP-ribosyltransferase	Nuclear
		Deacetylase	
SIRT7	44.8	Deacetylase	Nucleolar

Among all mammalian sirtuins, SIRT1 has been the most extensively studied. Studies of the other mammalian sirtuins have uncovered a variety of substrates, interacting partners, and biological relevance in diverse cellular processes (Donmez & Guarente, 2010; Michan & Sinclair, 2007; Nakagawa & Guarente, 2011).

## Sirtuins and neurodegeneration

Neurodegenerative disorders primarily affect the elderly population. With the increase in life expectancy, these disorders are contributing to a tremendous increase in health expenditures. The economic cost of Alzheimer's disease (AD) is enormous, and is expected to grow rapidly as more people live to a greater age with more serious impairments. In the US, for example, the Alzheimer's Association estimates that the cost of providing care for AD patients is $183 billion per year, as of 2011. If present trends continue, this cost is projected to grow to $1.1 trillion per year (in 2011 dollars) by 2050 – an overwhelming economic burden. Therefore, it is urgent to develop novel strategies for therapeutic intervention in these diseases.

A common hallmark of several neurodegenerative disorders is the loss of specific populations of neuronal cells and the presence of abnormal protein inclusions containing specific misfolded proteins (Mattson & Magnus, 2006). However, the precise molecular mechanisms involved in the neurodegenerative process remain unclear.

Aging is the major risk factor for the development of neurodegenerative disorders. While aging has been being recognized as a strong disease modifier, this pathway was not fully amenable for therapeutic manipulation until the discovery of sirtuins (de Oliveira et al, 2010, 2012). Different sirtuins have been recently found to modulate neurodegeneration and the toxicity associated with different proteins such as α-synuclein, huntingtin, tau or Aβ peptide (Donmez, 2012).

**Glossary**

**Aging**

Complex process of involving the accumulation of changes in a living organism over time.

**Alpha-secretase**

Enzymes that are members of the ADAM (a disintegrin and metalloprotease domain) family and cleave amyloid precursor protein (APP).

**Alzheimer's disease**

The most common age-related neurodegenerative disorder caused by neuronal loss resulting in dementia.

**Amyloid beta**

Peptide of 36–43 amino acids, generated by the sequential proteolytic cleavage of amyloid precursor protein, of which accumulation causes amyloid plaques in AD.

**Calorie restriction**

A dietary regimen that restricts calorie intake and was shown to decelerate biological aging process in model organisms.

**Deacetylases**

A class of enzymes that remove the acetyl group (O=C–CH_3_) from an ε-*N*-acetyl lysine amino acid.

**Extra-chromosomal rDNA circles**

Self-replicating circles of ribosomal DNA found in some strains of yeast and thought to contribute to their aging.

**Fatty acid oxidation**

The metabolic pathway in which fats are metabolized to release energy. It does not become a major source of energy until the animal's carbohydrate sources are completely utilized.

**Huntington's disease**

A genetic neurodegenerative disorder caused by an autosomal dominant mutation in the Huntingtin (IT-15) gene leading to a disorder characterized by involuntary movement and psychiatric alterations.

**Lewy bodies**

Intracellular proteinaceous accumulations primarily composed alpha-synuclein and ubiquitin that are observed in PD and other disorders known as synucleinopathies.

**Mitochondrial biogenesis**

A process by which new mitochondria are generated in the cell as a result of different stimuli.

**Myelination**

The formation of a sheath around the axon of a neuron by myelin, which is an electric insulating material called myelin.

**Oligodendrocyte**

Type of glial cells in the brain that form the myelin sheath around the axons in brain.

**Parkinson's disease**

The second common age-related neurodegenerative disorder known primarily as a movement disorder due to the loss of dopaminergic neurons and the deposition of protein inclusions known as Lewy bodies.

**Resveratrol**

A natural phenol produced by certain plants when attacked by bacteria or fungi.

**Tau**

Abundant protein in the central nervous system that stabilizes microtubules. Mutations of tau cause dementias such as frontotemporal dementia and AD.

### SIRT1 and neurodegenerative diseases

SIRT1 is ubiquitously expressed in all tissues including the brain. SIRT1 is known as a nuclear protein, which is predominantly expressed in neurons (Ramadori et al, 2008). However, SIRT1 has both nuclear import and export sequences and was found present in the cytosolic fraction of the mouse brain (Tanno et al, 2007).

**Figure 1:**
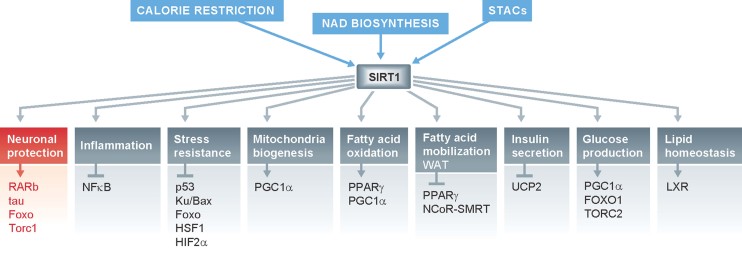
The targets and interacting partners of SIRT1 SIRT1 has many targets that play roles in different molecular pathways including neuronal protection, inflammation, stress resistance, mitochondrial biogenesis, fatty acid oxidation and mobilization, insulin secretion, glucose production and lipid homeostasis. SIRT1 is activated by CR, NAD biosynthesis and small molecule sirtuin activators (STACs).

SIRT1 binds to and deacetylates a number of important transcription factors (Fig 1) such as peroxisome proliferator-activated receptor alpha (PPARα), PPAR gamma coactivator 1 alpha (PGC-1α) and LXR in liver, NF-kB, p65, retinoic acid receptor beta (RARβ) and tau in brain, PPARγ in white adipose tissue, PGC1α and the forkhead box, subgroup O (FOXO) family of transcription factors in skeletal muscle, pancreas and brain (Donmez, 2012). Since FOXO transcription factors are involved in different molecular pathways such as neuronal protection, stress resistance and glucose production, SIRT1's involvement in the mechanisms is partly through FOXO (Fig 1; Salminen et al, 2008). SIRT1 activity is regulated by NAD^+^/NADH/NAM, by its protein levels, phosphorylation and by DBC1 and AROS proteins (Haigis & Sinclair, 2010).

Energy deprivation increases nicotinamide adenine dinucleotide (NAD^+^) levels and activates the NAD^+^-dependent deacetylase activity of SIRT1. Activated AMPK and SIRT1 converge by activating PGC-1α via phosphorylation and deacetylation, respectively, to induce mitochondrial biogenesis and fatty acid oxidation. Cross talk in this pathway occurs because AMPK activity increases NAD^+^, and SIRT1 also activates AMPK (Canto & Auwerx, 2010; Imai, 2009).

#### SIRT1 and Alzheimer's disease

Modulation of SIRT1 levels and/or activity has been recently shown to have beneficial effects in different models of AD, the most common and one of the most devastating age-related neurodegenerative diseases, causing severe cognitive and behavioural deficits.

Calorie restriction was shown to reduce amyloid plaques, one of the typical pathological hallmarks of the disease, in the APPswe/PS1dE9 mouse model (Qin et al, 2006). The researchers identified the mechanism as the activation of SIRT1 as a result of CR. In an *in vitro* model, SIRT1 protects against microglia-dependent amyloid-beta (Aβ) toxicity through inhibiting NF-κB signaling (Chen et al, 2005).

Pharmacological studies also indicate the protective role of SIRT1. Mice inducibly overexpressing a toxic coactivator of cyclin-dependent kinase 5 (CDK5), p25, were shown to display massive degeneration of forebrain with features of AD. In this particular mouse model, resveratrol reduced neurodegeneration in the hippocampus, prevented learning impairment and decreased the acetylation of the known SIRT1 substrates PGC-1alpha and p53. Furthermore, injection of SIRT1 lentivirus in the hippocampus of p25 transgenic mice conferred significant protection against neurodegeneration (Kim et al, 2007).

Overexpression studies showed that SIRT1 is protective against Aβ plaque formation in animal models. In a recent study, the APPswe/PS1dE9 mouse model, in which plaques form at around 3 months of age was used (Donmez et al, 2010). SIRT1 was overexpressed or deleted in APPswe/PS1dE9 mice by crossing with SIRT1-overexpressing or SIRT1-brain-specific-knockout mouse. Overexpression of SIRT1 reduced Aβ production and Aβ plaques, whereas deleting SIRT1 increased Aβ levels. SIRT1 deacetylates RARβ and activates ADAM10 (α-secretase) transcription, leading to upregulated APP processing by α-secretase, resulting in reduced production of Aβ. SIRT1 overexpression in this mouse model also improved learning and memory deficits and deletion of SIRT1 worsened these phenotypes (Donmez et al, 2010). The widely accepted β-amyloid hypothesis suggests that Aβ is the major etiological agent of AD pathology. Broad therapeutic strategies have therefore been focused on the inhibition of accumulation and aggregation of neurotoxic Aβ. Thus, SIRT1 might constitute an option for modulating the levels of Aβ.

Hyperphosphorylation of the microtubule-interacting protein tau, another major player in AD, can result in the self-assembly of tangles of paired helical filaments and straight filaments, which are involved in the pathogenesis of AD and other tauopathies. Degradation of phosphorylated tau improves cognitive function and reduces neuronal cell death in mice; however, when tau is acetylated by the histone acetyltransferase, p300, the breakdown of tau is inhibited (Min et al, 2010). SIRT1 was shown to deacetylate tau and consequently reduce its level; conversely, SIRT1 inhibition led to the opposite effect – increasing the levels of tau and exacerbating the accumulation of pathogenic forms of phosphorylated tau.

In addition to the findings in support of the protective effects of SIRT1 on neurodegeneration, there are also contradictory studies reporting the opposite effect. In this respect, it was shown that SIRT1 inhibition reduces IGF-1/IRS-2/Ras/ERK1/2 signalling and protects neurons (Li et al, 2008).

#### SIRT1 and Parkinson's disease

To date, there is a limited number of studies linking Parkinson's disease (PD) and SIRT1. PD is an age-associated neurodegenerative disorder primarily known as a motor disorder due to the loss of dopaminergic neurons from the substantia nigra in the brain. PD is also known for the accumulation of protein inclusions known as Lewy bodies, that are mainly composed of misfolded α-synuclein (Spillantini et al, 1997), a protein whose function remains obscure. Recently, overexpression of SIRT1 in animal and cell models of PD was shown to suppress the formation of α-synuclein aggregates by activating molecular chaperones (Donmez et al, 2012).

Resveratrol was shown to have a protective effect against α-synuclein-induced toxicity in SK-N-BE cells (Albani et al, 2009). In addition, dietary oxyresveratrol was found to prevent parkinsonian mimetic 6-hydroxydopamine neurotoxicity (Chao et al, 2008). In addition, CR or the use of 2-deoxy-d-glucose, a glucose analogue, was found to reduce the loss of dopaminergic neurons in mice and improves motor function (Duan et al, 2003).

The observations reported above suggest that SIRT1 might indeed constitute a target for intervention, but additional studies are required to unravel the molecular basis of protection. On the other hand, the existing studies did not demonstrate a protective role for sirtuins. For example, no protection was observed in an MPTP-induced PD model using SIRT1 transgenic mice (Kakefuda et al, 2009).

#### SIRT1 and Huntington's disease

Huntington's disease (HD) is an autosomal dominant neurodegenerative disorder characterized by a gradual and progressive loss of neurons, predominantly in the cortex and striatum leading to impairment in muscle coordination, cognitive decline and dementia. In a *Caenorhabditis elegans* model of HD, upregulation of SIRT1 or resveratrol treatment were shown to rescue neurons from injury induced by mutant huntingtin protein (HTT) (Parker et al, 2005). Expansions of CAG-trinucleotide repeats in the IT-15 gene that encodes for HTT, result in the production of HTT protein containing extended polyglutamine repeats that cause the misfolding and aggregation of the protein (Bates, 2003).

In a yeast model of HD, induction of Sir2 by oxidative stress and its activation reduce protein aggregation (Sorolla et al, 2011). On the other hand, in the N171-82Q transgenic mouse model of HD, there was no significant improvement in weight loss, motor performance, survival and striatal atrophy by using the SIRT1 activator resveratrol (SRT501-M; Ho et al, 2010). However, a recent study showed that brain-specific knockout of Sirt1 resulted in exacerbation of brain pathology in R6/2 mouse model of HD, whereas overexpression of Sirt1 improved survival, neuropathology and the expression of brain-derived neurotrophic factor (BDNF), which requires the presence of CREB-regulated transcription coactivator 1 (TORC1; Jeong et al, 2012a). Another group has also shown that Sirt1 prevents the mutant-HTT-induced decline in BDNF concentrations and the signaling of its receptor, TrkB and restores dopamine- and cAMP-regulated phosphoprotein, 32 kDa (DARPP32) concentrations in the striatum (Jiang et al, 2012). Sirt1 deacetylase activity is required for Sirt1-mediated neuroprotection in cell models of HD. Notably, it was shown that mutant HTT interacts with Sirt1 and inhibits Sirt1 deacetylase activity, which results in hyperacetylation of Sirt1 substrates such as forkhead box O3A (FOXO3a), thereby inhibiting its pro-survival function (Jiang et al, 2012).

Despite the great strides in understanding the molecular underpinnings of HD, no therapeutics are currently available that prevent progression of this devastating disease. However, in contrast to the protective effects of SIRT1 against HD, a phase 1 clinical trial is underway to treat HD with the highly specific SIRT1 inhibitor, EX-527 (Zhang et al, 2011). The outcome of the clinical trial will unravel whether inhibition of SIRT1 would be beneficial for the patients. Thus, these accumulating data suggest SIRT1 might constitute an attractive target for intervention in HD.

### SIRT2 and neurodegenerative diseases

HST2, the yeast orthologue of mammalian SIRT2, is upregulated by CR as well as oxidative stress and extends lifespan in yeast in a SIR2-independent manner (Lamming et al, 2005). In mammals, it is still unclear whether SIRT2 overexpression alters lifespan. SIRT2 is present primarily in the cytoplasm, co-localizes with microtubules and deacetylates the major component of microtubules, α-tubulin at lysine 40 (North et al, 2003). HDAC6 is a binding partner of SIRT2 but it is unclear whether SIRT2 deacetylates soluble or polymerized α-tubulin, and whether it can be formally considered a microtubule deacetylase.

SIRT2 transiently migrates to the nucleus during G_2_/M transition and deacetylates histone H4 at lysine 16, thereby modulating chromatin condensation during metaphase (Vaquero et al, 2006). In addition to α-tubulin and histone H4 substrates, SIRT2 deacetylates forkhead transcription factors of class O, FOXO1 and FOXO3 (Li et al, 2007). Since FOXO transcription factors are involved in multiple cellular processes, including DNA repair, cell cycle, apoptosis, metabolism and ageing, SIRT2 is also connected with these diverse pathways (Calnan & Brunet, 2008). SIRT2 deacetylates lysine residues in the catalytic domain of p300, a histone acetyltransferase. In addition, several proteins such as 14-3-3 β/γ and homeobox transcription factor 10 are binding partners but not deacetylation substrates of SIRT2 (Bae et al, 2004). The diversity in SIRT2 substrates and binding partners suggests a complex and diverse function for this sirtuin.

Several stimuli can activate autophagy and, notably, one of the main triggers is nutrient deprivation. In response to oxidative stress or serum deprivation SIRT2 releases FOXO1, which is then acetylated and binds to ATG7 and thus induces autophagy in the context of cancer (Zhao et al, 2010). Accordingly, it would be interesting to test whether SIRT2 also mediates autophagy through deacetylation of FOXO1 in the context of neurodegeneration. This hypothesis seems plausible concerning the stimulus for SIRT2 to release FOXO1 and making it available to activate autophagy. Interestingly, SIRT1 also plays a role in the regulation of autophagy through deacetylation of ATG5, 7 and 8 (Lee et al, 2008). It is widely accepted that oxidative stress is implicated in the pathogenesis of neurodegenerative diseases. SIRT2 elevates the expression of the antioxidant mitochondrial superoxide dismutase (MnSOD) due to its ability to deacetylate FOXO3 and consequently increase FOXO3 DNA-binding activity (Wang et al, 2007). Interestingly, the enzymatic activity of MnSOD is regulated by SIRT3 deacetylation in response to stress (Tao et al, 2010).

Another interesting link between SIRT2 and neurodegeneration is through NF-κB. NF-κB plays a pivotal role in regulating gene expression programs related to aging and inflammation, namely by inducing the expression of pro-inflammatory cytokines. Recent studies point to an association between chronic neuroinflammation and the exacerbation of several neurodegenerative diseases (Salminen & Kaarniranta, 2009). As the organism ages, NF-κB transcription is activated and may elicit neurodegeneration. SIRT2 has been reported to interact with p65, an NF-κB family member, in the cytoplasm and to deacetylate it at lysine 310 after stimulation with TNF-α (Rothgiesser et al, 2010). Interestingly, deacetylation of p65 by SIRT1 antagonizes NF-κB activity (Yeung et al, 2004).

SIRT2 was also associated with the aggregation process of proteins such as α-synuclein and huntingtin, involved in PD and HD, respectively. In addition, SIRT2 was indirectly associated with cellular processes implicated in the pathophysiology of neurodegenerative disorders, namely autophagy, oxidative stress and inflammation. The roles of SIRT2 in these neurodegenerative diseases will be discussed in the following sections.

#### SIRT2 in Parkinson's disease

Inhibition of SIRT2 function, either pharmacologically or genetically, was found to result in the rescue of α-synuclein toxicity in different *in vitro* and *in vivo* models of PD. In a human cell line model of α-synuclein inclusion formation, pharmacological inhibition of SIRT2 promoted the development of fewer and larger α-synuclein Lewy body-like inclusions. Interestingly, a smaller number of these inclusions that were larger in size, which was associated with the protective effects observed (Outeiro et al, 2007). This effect is consistent with the current view that larger aggregates may actually be protective, whereas smaller intermediates, known as oligomeric species, may constitute the toxic genus. Importantly, the protective effect of SIRT2 inhibition was further confirmed in rat primary midbrain cultures, where expression of A53T mutant α-synuclein caused selective loss of tyrosine hydroxylase (TH) positive cells, and also *in vivo*, in a Drosophila model of PD. The study by Outeiro and colleagues, using a variety of *in vitro* and *in vivo* models, provided the foundation for studies where inhibition of SIRT2 might prove beneficial. Although additional studies are still required, since the precise molecular mechanisms underlying SIRT2-mediated protection are still elusive, it was tempting to speculate that SIRT2 inhibition increases microtubule-dependent transport of putative neurotoxic α-synuclein oligomers to the nucleation aggregation site, which facilitates formation of large benign inclusions. Consistent with this idea, an interaction between microtubules and α-synuclein was reported by several groups (Alim et al, 2004; Iseki et al, 2000; Payton et al, 2001). One possibility is that the interaction between oligomeric α-synuclein and acetylated microtubules may be enhanced by inhibition of the microtubule deacetylases SIRT2 or/and HDAC6. Thus, genetic crosses between PD and SIRT2 brain-specific knockout mice will provide valuable insight into neuroprotective mechanism(s) and further report on the therapeutic potential of SIRT2 inhibition in PD.

Recently, it was shown that SIRT2 in the brain enhances 1-methyl-4-phenyl-1,2,3,6-tetrahydropyridine (MPTP)-induced nigrostriatal damage via deacetylating Foxo3a and activating Bim, a proapoptotic factor. By administering MPTP by a chronic regimen that causes apoptotic neuronal death to wildtype and SIRT2 KO mice (Bobrowska et al, 2012), it was shown that apoptotic neuronal death in SIRT2 KO mouse brain was prevented due to the low levels of Bim protein (Liu et al, 2012).

#### SIRT2 in Huntington's disease

In a fly model of HD, decreased levels of SIRT2 promoted the viability of photoreceptor neurons (Pallos et al, 2008). Pharmacological inhibition of SIRT2 resulted in neuroprotection in cellular and invertebrate models of HD through a negative regulation of sterol biosynthesis (Luthi-Carter et al, 2010).

In primary striatal neurons expressing a mutant HTT fragment, genetic or pharmacological inhibition of SIRT2 was associated with significant reduction of polyglutamine inclusions. The reduction of inclusion formation, which is believed to be dependent on microtubule transport, may represent a benign biomarker. Another major finding from this study is that this protective effect is intimately related to transcriptional regulation of genes controlling metabolism, including sterol and fatty acid biosynthesis, carbohydrate metabolism and purine metabolism (Luthi-Carter et al, 2010). More specifically, SIRT2 inhibition reduced sterol levels via decreased nuclear trafficking of sterol regulatory element-binding protein 2 (SREBP-2) and resulted in lower cholesterol levels. Notably, in the same model, manipulation of sterol biosynthesis at the transcriptional level mimicked SIRT2 inhibition, demonstrating that the metabolic effects are sufficient to diminish mutant HTT toxicity. Genetic manipulation of SREBP-2 expression levels and/or subcellular localization had no effect on the aggregation state of mutant HTT fragments. In follow-up studies, pharmacological inhibition of SIRT2 in wild type primary neurons resulted in SREBP-2 cytoplasmic retention, transcriptional downregulation of cholesterol biosynthetic genes and reduction of neuronal cholesterol (Taylor et al, 2011). The experiments were extended to Neuro-2a (N2a) neuroblastoma cells and to hippocampal slice cultures from wild type mice, where SIRT2 inhibition-dependent reduction of cholesterol levels was observed as well (Taylor et al, 2011).

In contrast, genetic ablation of SIRT2 was recently found to have no effect on tubulin acetylation in the brain or on cholesterol biosynthesis, failing also to modulate the progression of disease in the widely used R6/2 mouse model of HD (Bobrowska et al, 2012). While these findings are intriguing, the R6/2 model is known for displaying particularly aggressive phenotypes, so additional studies are required with other established animal models of HD in order to clarify the role of SIRT2 in the modulation of the disease.

Together, these results suggest a novel and attractive role of SIRT2 in regulation of neuronal metabolism, and specifically of cholesterol biosynthesis, that should be further investigated. Interestingly, the fact that the same small molecule is protective in PD and HD, two devastating disorders, is highly promising, suggesting it might also have a beneficial impact on other neurodegenerative diseases.

### Sirtuins and other neurodegenerative conditions

Sirtuins have also been implicated in other neurodegenerative conditions and in protection against axonal degeneration, a process that occurs in neurodegenerative diseases and peripheral neuropathies. In a mouse model of Wallerian degeneration (*Wld*^*s*^), the anterograde degeneration of transected axons is markedly delayed due to a mutation resulting in overexpression of a chimeric protein (Wld^s^) composed of the ubiquitin assembly protein Ufd2a and the nicotinamide adenine dinucleotide (NAD) biosynthetic enzyme Nmnat1 (Conforti et al, 2000). It was suggested that the activity of Nmnat1 alone (independent on Ufd2a) provides the axon-protective activity of the Wlds protein and that this is mediated by NAD production (Araki et al, 2004). Resveratrol- or NAD-pretreated neurons exhibited a decrease in axonal degradation after axon transection. Furthermore, knock down of SIRT1 or treatment with sirtinol blocked NAD-dependent axonal protection. Therefore, SIRT1 was proposed as the downstream effector in the Nmnat/NAD axonal protection activity. However, other groups suggested that other SIRT1-independent mechanisms underlie the Nmnat/NAD neuroprotective effect. It was reported that the degeneration of transected axonal segments could be prevented by NAD exogenous local application 24 h prior to axon transection. Furthermore, similar protective effects of NAD could be observed in axons exposed to NAD directly at the time of transection or even until 5 h after transection (Wang et al, 2005). This suggests that the NAD-dependent axon protection may be mediated primarily by its effect on local bioenergetics than through NAD-induced transcription and other nuclear events.

The role of SIRT2 in Wallerian degeneration has also been studied. In particular, the hypothesis that suppression of microtubule depolymerization delays axonal degeneration was tested, since the *Wld*^s^ phenotype shows a substantial resistance to microtubule depolymerizing drugs (Suzuki & Koike, 2007; Wang et al, 2000). The basal level of microtubule acetylation (stabilization) is increased in cultured cerebellar granule cells from *Wld*^s^ mice. SIRT2 overexpression abolishes microtubule hyperacetylation and resistance to axonal degeneration in these cells. Furthermore, SIRT2 knock-down enhances microtubule acetylation and resistance to axonal degeneration in wild-type cerebellar granule cells (Suzuki & Koike, 2007).

Several studies have also reported beneficial effects of SIRT1 and CR on prion-mediated neurodegeneration. In particular, the onset of prion disease is delayed by CR and in SIRT1 knockout mice fed *ad libitum* (Chen et al, 2008), through a process in which CR and SIRT1-mediated protection seem to be connected. In *C. elegans*, increased dosage of sir-2.1, the orthologue of mammalian SIRT1, and resveratrol, were both able to reverse neurotoxicity induced by mutant prion protein (PrP) (Bizat et al, 2010). More recently, increased SIRT1 levels were found to regulate prion-induced neuronal cell death, possibly through induction of autophagy (Jeong et al, 2012b; Seo et al, 2012). Together, these studies highlight the complexity of SIRT1-mediated protective effects and suggest that it might be possible to target this sirtuin as a therapeutic strategy in prion diseases.

## Sirtuins in neuronal differentiation

SIRT1 deacetylates a series of transcription factors, such as p53, FOXOs, Ku70 or NF-κB, thereby regulating important processes such as cell differentiation, survival, tumourigenesis, stress resistance and metabolism. SIRT1 was also directed implicated in neural stem cell (NSC) biology, as it was found to be upregulated in embryonic NSCs exposed to oxidative conditions *in vitro* (Prozorovski et al, 2008). Interestingly, SIRT1 is required to induce differentiating NSCs to adopt an astrocytic fate under oxidative stress. While the use of oxidizing agents to uncover the effects of SIRT1 on NSCs raises questions about the physiological relevance, it also raises the attractive possibility that this mechanism might be in place during aging or age-associated neuropathological conditions, potentially contributing to brain repair. Furthermore, SIRT1 was found to positively modulate the Wnt signalling pathway (Holloway et al, 2010), which is deeply implicated in neural progenitor proliferation and neurogenesis in the hippocampus and subventricular zone (Lie et al, 2005; Qu & Shi, 2009; Sun et al, 2007). Thus, it will be crucially important to further investigate how SIRT1 may act upon different signalling pathways to regulate the fate of NSCs, as this may open new perspectives for therapeutic intervention.

The levels of SIRT2 in the brain increase with the developmental timing of myelination and increase as oligodendrocyte precursor cells differentiate into oligodendrocytes (Li et al, 2007). One possibility is that SIRT2 might act through FoxO1 and FoxO3 to regulate their transcriptional activities under conditions of changing energy availability or oxidative stress (Paik et al, 2009; Renault et al, 2009; Wang et al, 2007), key aspects in most, if not all, neurodegenerative conditions.

The potential roles of the other sirtuins in neuronal differentiation are currently less clear and, therefore, demand further investigation. Due to their mitochondrial localization (Fig 1), SIRT3, SIRT4 and SIRT5 might also interfere with NSC differentiation by modulating energy metabolism, but this needs to be explored further in future studies.

In summary, the emerging roles of sirtuins in neuronal differentiation and regeneration hold promise as strategies for intervention in neurological conditions.

## Conclusions

The precise functions of sirtuins are still unclear but they seem to be important players also in age-associated neurodegenerative disorders. Therefore, elucidating the molecular roles of sirtuins may enable the development of novel strategies for intervention in this type of disorders. Despite recent controversy on the role of sirtuins in lifespan extension, their actions on substrates known to play important roles in fundamental cellular processes related to the health status of the organisms, such as FOXO transcription factors, α-tubulin, PEPCK1 and NF-κB, indicate that they must be central players in the biology of cells and organisms. In addition, the connection between sirtuins and CR also warrants further investigation on the precise role of sirtuins. The latest report on CR in rhesus monkeys raises doubts on the lifespan benefits, but still suggests an overall healthspan effect might be attained (Mattison et al, 2012). Therefore, it might still be possible to develop ways for mimicking CR benefits by modulating sirtuin function.

The opposing effects of manipulating SIRT1 or SIRT2 activities in diseases such as PD and HD underscores the distinct nature and functions of these enzymes. This is not surprising given the fact that they are localized in different subcellular locations, and clearly demonstrates that SIRT1 and SIRT2 are remarkably different enzymes despite sharing the catalytic domain as a common structure. Therefore, inhibiting SIRT2 while activating SIRT1 seems to be beneficial for the organism against certain age-associated diseases. One possibility is that deletion or inactivation of SIRT2 could lead to activation of SIRT1. If so, it may still be desirable to activate SIRT1 to treat PD.

**Pending issues**

Elucidation of the functions of SIRT1 and SIRT2 homologues (SIRT2-7) in the mammalian brain.

Analysis of the roles of sirtuins in neurodevelopmental disorders.

Identification of the therapeutic potential of SIRT1 and SIRT2 against neurodegenerative disorders using different disease models.

Development of specific activators/inhibitors of SIRT1/SIRT2 for therapeutic intervention in neurodegenerative disorders.

**Figure 2:**
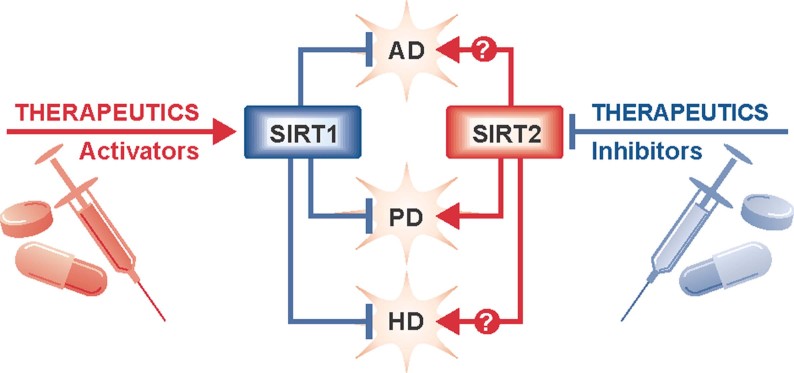
SIRT1 and SIRT2 in neurodegeneration As a result of numerous publications, SIRT1 seems to have protective effects against AD, PD and HD. Therefore, activating SIRT1 could be beneficial against these diseases. On the other hand, deletion of SIRT2 seems to be protective against PD. Because there is conflicting data regarding the effect of SIRT2 on HD and lack of data regarding the effect of SIRT2 on AD, the roles of SIRT2 in HD and AD remain unclear.

Pharmacological inhibitors or activators of sirtuins are attractive candidates as modulators of age-associated disorders, such as those covered herein (Fig 2). Nevertheless, it will be essential to develop highly selective molecules that enable the targeting of specific sirtuins, as their effects may, under some circumstances, overlap due to cross talk between different family members. In addition, understanding the molecular mechanisms underlying the protective role of all sirtuins in different organs could bring us closer to the identification of novel drug targets, which could be used to design new and more successful therapies and possibly even delay normal aging.
